# Retrospective analysis of 261 autopsies of penetrating cardiac injuries with emphasis on sociodemographic factors

**DOI:** 10.1038/s41598-023-38756-9

**Published:** 2023-07-18

**Authors:** Andres Isaza-Restrepo, Andrea Donoso-Samper, Elkin Benitez, Juan Sebastian Martin-Saavedra, Asdhar Toro, Daniel Felipe Ariza-Salamanca, Nora Arredondo, Nicolas Molano-Gonzales, Angela Maria Pinzon-Rondon

**Affiliations:** 1 Medical and Health Sciences Education Research Group, School of Medicine and Health Sciences, Universidad del Rosario, Bogotá, 111221 Colombia; 2 Surgery Department, School of Medicine and Health Sciences, Universidad del Rosario, Bogotá, 111221 Colombia; 3 Department of Neurology, Massachusetts General Hospital, Boston, MA USA; 4 Instituto Nacional de Medicina Legal y Ciencias Forenses, Bogotá, 111711 Colombia; 5 Clinical Research Group, School of Medicine and Health Sciences, Universidad del Rosario, Bogotá, 111221 Colombia

**Keywords:** Cardiology, Health policy, Prognosis, Public health

## Abstract

Penetrating cardiac injuries (PCIs) are highly lethal and several factors are related to its incidence and mortality. While most studies focus on characterizing patients who arrived at a medical facility alive and exploring the relationship between the degree of heart compromise and mortality, our study delved deeper into the topic. This study analyzed 261 autopsy reports from 2017 in Bogotá, Colombia, and characterized the factors surrounding PCI incidence and mortality while emphasizing the role of sociodemographic variables. Of these cases, 247 (94.6%) were males with a mean age of 29.19 ± 9.7 years. Weekends, holidays, and late hours had the highest incidence of PCIs. The victims' deaths occurred at the scene in 66 (25.3%) cases, and 65.1% of the victims died before receiving medical care. Upon admission, patients with vital signs were more likely to have been transported by taxi or a private vehicle. Two or more compromised cardiac chambers, increased time of transportation, trauma occurred in the city outskirts, and gunshot wounds were related to increased mortality. Our data is valuable for surgeons, health system managers, and policy analysts as we conducted a holistic assessment of the anatomical and sociodemographic factors that are closely associated with mortality following a PCI. Surgeons must recognize that PCIs can occur even when the entrance wound is outside the cardiac box. Reinforcing hospital infrastructure in the outskirts and improving the availability, accuracy, and response time of first responders may lead to improved patient mortality rates.

## Introduction

All types of injuries are responsible for the death of approximately 5 million people worldwide every year. Homicide is defined in the International Classification of Crime as "unlawful death inflicted upon a person with the intent to cause death or serious injury"^1^. In 2017, the global homicide rate was 6.1 per 100,000 population. However, there is a disparity in the rates between continents, with Asia having a rate of 2.3 per 100,000 and America having a rate of 17.2 per 100,000^2,3^. In Colombia, homicide is the leading cause of death among 15 to 29-year-olds, with data indicating 41.28 cases per 100,000 in 2017 and 38.8 cases per 100,000 in 2019^3^.

Colombia, a high-middle-income country with a long history of violence, has seen elevated homicide rates throughout its history due to strife between different armed actors, with rates increasing to 93 per 100,000 in 1993^4,5^. Recent agreements between the armed groups and the state have decreased violence. Nonetheless, Colombia still experiences high political tension that creates uncertainty regarding violence and homicide^5^. Nevertheless, there have been improvements over time.

Several factors are associated with the incidence of this type of trauma, including organized crime, drug trafficking, socio-demographic factors, low levels of social integration, interpersonal conflicts, drug and alcohol abuse, unemployment, income inequality, unequal access to education and health services, poor housing, mental health problems, and the failure to establish robust security and justice systems^2,6–9^.

Penetrating cardiac injuries (PCIs) are among the most lethal injuries as they are intended to cause death, and although they constitute a challenge for the trauma surgeon, the opportunity and efficiency for his attention can reflect the quality and response capacity of a system to trauma. Nearly 90% of patients with a PCI die due to cardiac tamponade or hemorrhagic shock before reaching a hospital facility^5^. In high-income countries, some cities report gunshot wounds (GSW)^10–13^ are the most frequent PCI mechanism, while others found stab wounds (SWs)^14,15^ are the most frequent; in low to middle-income countries PCIs are mainly caused by SWs. For instance, in a cases series in Bogotá, 93% of PCI patients arriving at a hospital between 1999 and 2009 had SWs^16^. Similarly, in another hospital case series in Medellin between 1997 and 1999, SWs accounted for 95% of PCIs^17^.

Regarding anatomical factors and the site of injury, any thoracic lesion within the limits of the cardiac box should be considered high risk for PCI^15^. However, unlike injuries to other organs, the immediate prognosis in PCI is affected by factors unrelated to the degree of injury, such as the quality of prehospital care, transfer time, characteristics of the injury, associated injuries, and prior use of psychoactive substances^16–19^. Given that the characteristics of the different series of PCI cases that reach medical and surgical care, around 10% of the total, have not varied substantially over time, therefore, to understand the high mortality rate associated with PCIs it is essential to characterize and study the socio-demographic, anatomical, and circumstantial aspects surrounding the 90% of cases that result in death due to PCIs.

To advance in the knowledge of the factors mentioned above, in this research, we analyzed the anatomical and sociodemographic factors of 261 cases perished by PCIs reported by the Colombian National Institute of Legal Medicine and Forensic Sciences (INMLCF) in 2017. Furthermore, we contrasted the results to the ones gathered by Pedraza et al.^20^ in 2007. Our objective was to track how factors surrounding homicide by PCIs changed from 2007 to 2017 and to understand how geographical and anatomical sites of injury impact the prognosis and mortality of these patients.

## Results

### 2017 Rresults

#### General characterization

Table 1 presents the results of demographic details, homicide characteristics, and medical attention received for a total of 261 PCI cases in 2017. 248 (94.6%) of these cases were males, and the mean age was 29.19 ± 9.7 years. Only 58 (36.2%) of the 160 registered an occupation status as employed, and of the 85 cases with reported literacy, 48 (57.4%) had a high school diploma.

**Table 1:** Demographic Characteristics, Homicide Characteristics and Medical Attention Characteristics of 2017.

Victim’s demographic characteristics	n = 261 (100%)
	n = 261
Male, sex	247 (94.6)
Age in years, mean(SD)	29.19 (9.7)
Occupation	n = 160
Employed	58 (36.2)
Independent	47 (29.3)
Unemployed	31 (19.3)
Other	24 (15)
Education	n = 85
Elementary	20 (23.5)
High school	48 (56.4)
University	16 (18.8)
None	1 (1.1)
Homicide characteristics	n = 261 (100%)
Aggressor	n = 89
Unknown	44 (49.4)
Known	35 (39.3)
Criminal Groups	9 (10.1)
Authority	1 (1.1)
Motive	n = 101
Fight	54 (53.4)
Robbery	22 (21.7)
Revenge	20 (19.8)
Family Violence	3 (2.9)
Other	2 (1.9)
Wound mechanism	n = 261
GSW	116 (44.5)
SW	145 (55.5)
Victims drug consumption,	n = 261
Alcohol	140 (53.6)
Others	12 (2.5)
Non-consumption	109 (41.7)
	n = 261
Trauma on holiday’s, n(%)	28 (10.7)
Medical attention	n = 261 (100%)
Death Place, n (%)	n = 261
Transportation	104 (39.8)
Medical facility	91 (34.9)
The scene	66 (25.3)
Transportation mean, n(%)	n = 242
Police vehicle	110 (45.4)
Private vehicle	39 (16.1)
Taxi	22 (9.1)
Ambulance	3 (1.2)
Other	2 (0.8)
Not transported	66 (27.2)
	n = 139
Time Wound to the ER in minutes, mean (SD)	23.8 (16.77)
People arriving a medical facility, n(%)	n = 261
Yes	195 (74.7)
No	66 (25.2)
Admission Vital Signs, n(%)	n = 195
Yes	51 (19.5)
No	144 (55.2)
Death on the scene	66 (25.3)
Type of medical attention, n(%)	n = 261
No medical attention	168 (64.3)
Surgical care	51 (19.5)
Emergency room care	40 (15.3)
Prehospital care	2 (7.6)
Initial Medical Center Level, n(%)	n = 261
1st (Lowest)	24 (9.2)
2nd	32 (12.3)
3rd	120 (45.9)
4th (Highest)	19 (7.2)
Death on the scene	66 (25.2)
Definitive medical center level, n(%)	n = 261
1st(Lowest)	17 (6.5)
2nd	29 (11.1)
3rd	126 (48.2)
4th (Highest)	23 (8.8)
Death on the scene	66 (25.2)
	n = 261
Referral to another hospital, n(%)	10 (5.1)
	n = 172
Time wound-death in minutes, mean (SD)	115.1 (681.9)

SD, standard deviation; GSW, gun-shot wounds; SW, stab wounds; ER, emergency room.

#### Sociodemographic characterization

**Figure 1:**
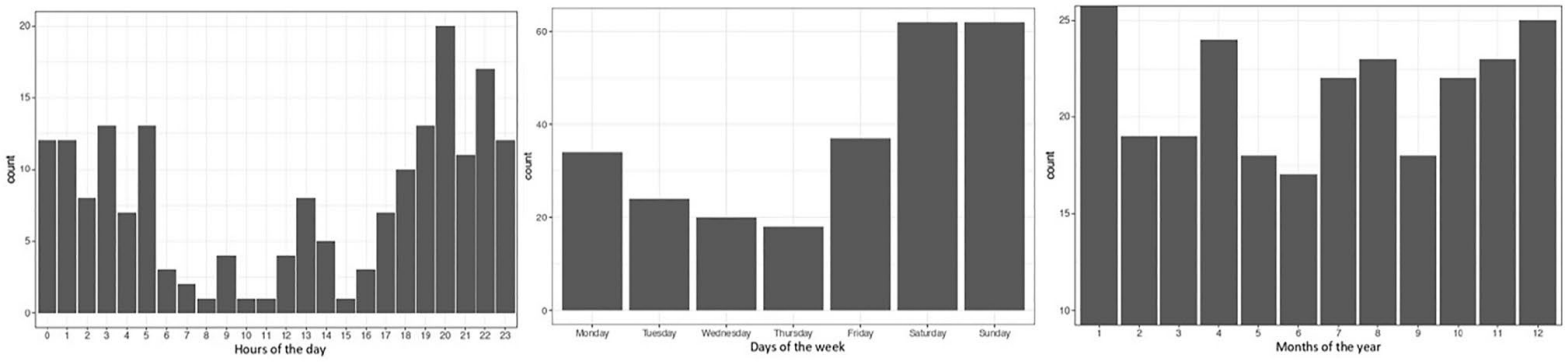
Time analysis on the frequency of penetrating cardiac injuries: (**A**). Hours of the day. (**B**). Days of the week. (**C**). Months of the year.

The months with the highest PCI rates were January with 27 (10.5%) cases, December with 25 (9.7%) cases, and April with 24 (9.3%) cases. Saturday and Sunday were the days with the highest rates, with 62 (24.1%) cases each, and special days such as holidays, soccer games, and music festivals accounted for 28 (10.7%) cases. Homicides were most common at 20:00 h, with a total of 20 (10.6%) victims, and at 22:00 with 17(9.0%) cases (Fig. 1). Of the 101 registered homicide motives, 54 (53.4%) were due to brawls.

We collected 242 transportation records: the police transported 110 (45.4%) and ambulances transported 3 (1.2%) cases. Transportation time was available in 135 records, with an average time of 24 ± 16 min. Of the total number of cases, victims died at the scene, 104 (39.8%) died while being transported, and 91(34.8%) died at the medical facility, meaning that 65.1% died before receiving medical care.

The injuries were SWs in 145 (55.5%) cases, and GSWs in 116 (44.5%) cases. Of the total cases, 195 (74.7%) arrived at a medical facility, 51 (19.5%) cases registered vital signs upon arrival, and patients in an agonic phase received medical care as well. Overall, 168 (64.3%) cases did not receive medical attention, while 2 (7.6%) received prehospital care, 40 (15.3%) received emergency room care, and 51 (19.5%) underwent surgery. Most patients, 120 cases (45.9%), arrived at third-level medical centers (locally, the highest complexity hospital is fourth level).

First-level institutions received 24 (9.2%) patients and referred 10 (5.1%) to another hospital. Of all patients that reached a medical facility, 51 (19.4%) had registrable vital signs, and 91 (34.7%) received medical attention according to the Ivatury trauma scale. The time from the injury to the declaration of death was registered in 117 cases (49.1%) and accounted for 114.5±680 min. Finally, 140 (53.6%) victims had consumed alcohol before the homicide.

### Trauma characteristics

**Figure 2:**
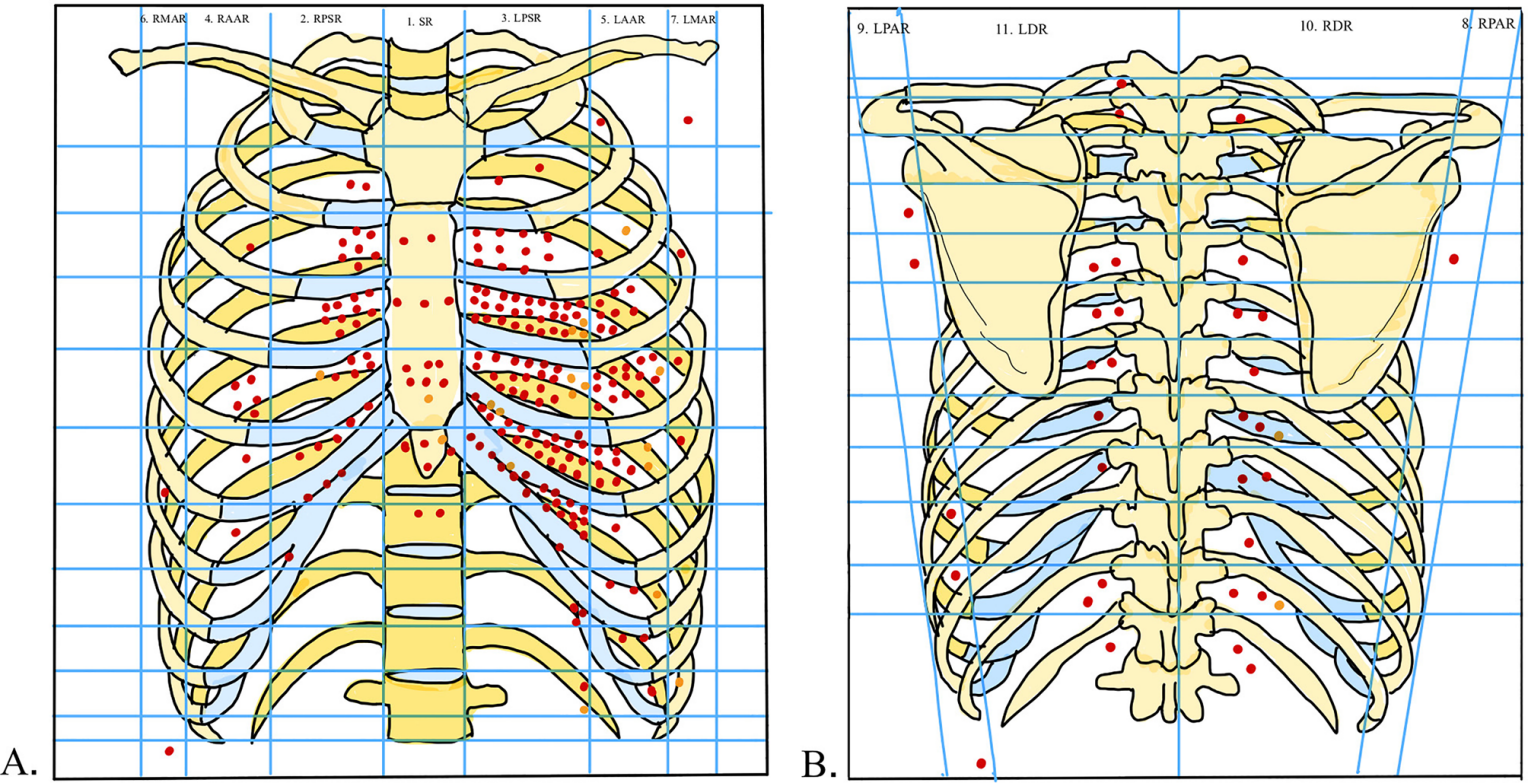
Topographic analysis on the entrance wounds with heart compromise. Sternal region (SR), right parasternal region (RPSR), left parasternal region (LPSR), right anterior axillary region (RAAR), left anterior axillary region (LAAR), right middle axillary region (RMAR), left mid-axillary region (LAMR), right posterior axillary region (RPAR), left posterior axillary region (LPAR), right dorsal region (RDR), left dorsal region (LDR). Image taken and modified of Netter 2018.

The mean number of thoracic wounds was 2.6 (SD, 4.15), and the PCIs were within the limits of the cardiac box in 202 (77.1%) cases. The injuries compromised the right ventricle in 153 (60.4%) victims and the right atrium in 61 (24.1%). Overall, 158 (62.4%) victims had one injured chamber. According to the Injury Scoring Scale—American Association for the Surgery of Trauma^21^, 135 (52.1%) cases had IV-grade injuries, and 117 (45.1%) had V-grade injuries. We illustrated PCIs entrance wounds in Fig. 2 and described them in Table 2.

**Table 2:** Heart trauma characteristics.

Heart Trauma characteristics	n = 261	100%
Number of thorax wounds (mean ±SD), y	2.6 (± 4.15)	
Thorax wounds with heart compromise in the precordium, n(%)	202	77.1%
Abdomen wounds with heart compromise, n(%)	7	2.6%
Compromised Cardiac Chambers, n(%)	n = 253	n = 253
One chamber	158	62.4%
Two chambers	86	33.9%
Three chambers	9	3.5%
Chamber compromised	n = 253	n = 253
Right atrial wound, n(%)	61	24.1%
Left atrial wound, n(%)	20	7.9%
Right ventricular wound, n(%)	153	60.4%
Left ventricular wound, n(%)	123	48.6%
OIS-ASST Classification, n(%)	n = 259	n = 259
I	3	1.15%
II	0	0.0%
III	2	0.7%
IV	135	52.1%
V	117	45.1%
VI	2	0.7%
Associated lesions	n = 261	100%
Face and head wounds, n(%)	74	28.2%
Neck wounds, n(%)	44	16.7%
Abdomen wounds, n(%)	54	20.6%
Extremities wounds, n(%)	111	42.3%
Victims with other compromised organs(%)	206	78.6%
Lung injury, n(%)	173	66.0%
Diaphragm injury, n(%)	76	29.0%
Liver or spleen injury, n(%)	67	25.5%
Gastrointestinal injury, n(%)	34	12.9%
Aorta’s injury, n(%)	28	10.6%
Injury of other thorax vessels, n(%)	25	9.5%
Spine injury, n(%)	21	8.0%
Cava vein injury, n(%)	13	4.9%
	10	3.8%

OIS-ASST, Organ injury scaling. IV: Thoracic vascular, lung, cardiac, and diaphragm.

### Associated Lesions

All the cases had associated lesions, Table 2 summarizes the results. The most common associated injured body segments were the extremities in 111 (42.3%) cases, followed by the head or face in 74 (28.2%) cases, the neck in 44 (16.7%) cases, and the abdomen in 54 (20.6%) cases. Interestingly, in 7 cases, abdominal injuries compromised the heart. Internal organs with associated lesions were as follows: the lungs in 173 (66%) cases, the diaphragm in 76 (29%) cases, the liver or the spleen in 67 (25.5%) cases, the gastrointestinal tract in 34 (12.9%) cases, the great vessels, aorta and cava in 41 (15.5%) cases, other thorax vessels in 25 (9.5%) cases, and the airway in 10 (3.8%) cases.

### Bivariate analysis

We conducted a bivariate analysis of vital signs upon admission and injury mechanism. Table 3 summarizes the results.

**Table 3:** Bivariate analysis.

Admission with vital signs				
	Death on the Scene n = 66 (25.3%)	Admitted without vital signs n = 144 (55.2%)	Admitted with vital signs n = 51 (19.5%)	*p *value
Wound mechanism, n(%)				
GSWs	34 (51.5%)	69 (47.9%)	13 (25.4%)	0.008
SWs	32 (48.4%)	75 (52.0%)	38 (74.5%)	
Type of medical attention, n(%)				
Prehospital care	0 (0.0%)	2 (0.01%)	0 (0.0%)	< 0.001
Medical care	0 (0.0%)	24 (16.6%)	16 (31.4%)	
Surgical care	0 (0.0%)	16 (11.1%)	35 (68.6%)	
Death before arrival to the ER	66 (100%)	102 (70.8%)	0 (0.0%)	
Transportation mean, n(%)				
Ambulance	0 (0.0%)	3 (2.2%)	0 (0.0%)	< 0.001
Taxi	0 (0.0%)	13 (9.7%)	9 (21.4%)	
Police vehicle	0 (0.0%)	93 (69.4%)	17 (40.4%)	
Private vehicle	0 (0.0%)	25 (18.6%)	14 (33.3%)	
Other	0 (0.0%)	0 (0.0%)	2 (4.7%)	
No transportation	66 (100%)	0 (0.0%)	0 (0.0%)	
Initial medical center level, n (%)				
1st(Lowest)	0 (0.0%)	13 (9.0%)	11 (21.5%)	< 0.001
2nd	0 (0.0%)	25 (17.3%)	7 (13.7%)	
3rd	0 (0.0%)	95 (65.9%)	25 (49.0%)	
4th (Highest)	0 (0.0%)	11 (7.6%)	8 (15.6%)	
Death on the scene	66 (100%)	0 (0.0%)	0 (0.0%)	

Injury mechanism				
	GSWs n = 112 (46.1%)	SWs n = 131 (53.9%)		*p *value
Motive, n(%)				
Fight	14 (32.5%)	40 (68.9%)		0.001
Robbery	11 (25.5%)	11 (18.9%)		
Revenge	15 (34.8%)	5 (8.6%)		
Family violence	1 (2.3%)	2 (3.4%)		
Other	2 (4.6%)	0 (0.0%)		
Victims drug consumption, n (%)				
Alcohol	45 (38.7%)	95 (65.5%)		< 0.001
Non consumption	65 (56.0%)	44 (30.4%)		
Others	6 (5.1%)	6 (4.1%)		
Time wound-death (mean ±SD), min	30.3 (± 52.1)	204.0 (± 969.3)		0.001
Death place, n (%)				
Scene	34 (29.3%)	32 (22.1%)		0.045
Transportation	51 (44.0%)	53 (36.5%)		
Medical facility	31 (26.7%)	60 (41.4%)		

SD, Standard deviation; GSW, Gun-shot wounds; SW, Stab wounds; ER, Emergency room.

#### Vital signs upon admission

Most of the patients who arrived with vital signs at a medical facility were transported by taxi or a private vehicle; from those cases, 38 (74.5%) had suffered a SW, and 27 (68,6%) had a surgical intervention upon arrival.

#### Injury mechanism

SWs were the most common mechanism if the victim was under the influence of alcohol (*p* < 0.001) and if the homicide occurred during a brawl (*p* = 0.001). Victims who sustained SWs were more likely to die in a medical facility, with 60 cases (41.4%) compared to 53 cases (36.5%) during transportation and 32 cases (22.1%) at the scene of the incident. On the other hand, GSW victims were more likely to die during transportation 51(44.0%) (*p* = 0.04).

## Georeferencing

**Figure 3:**
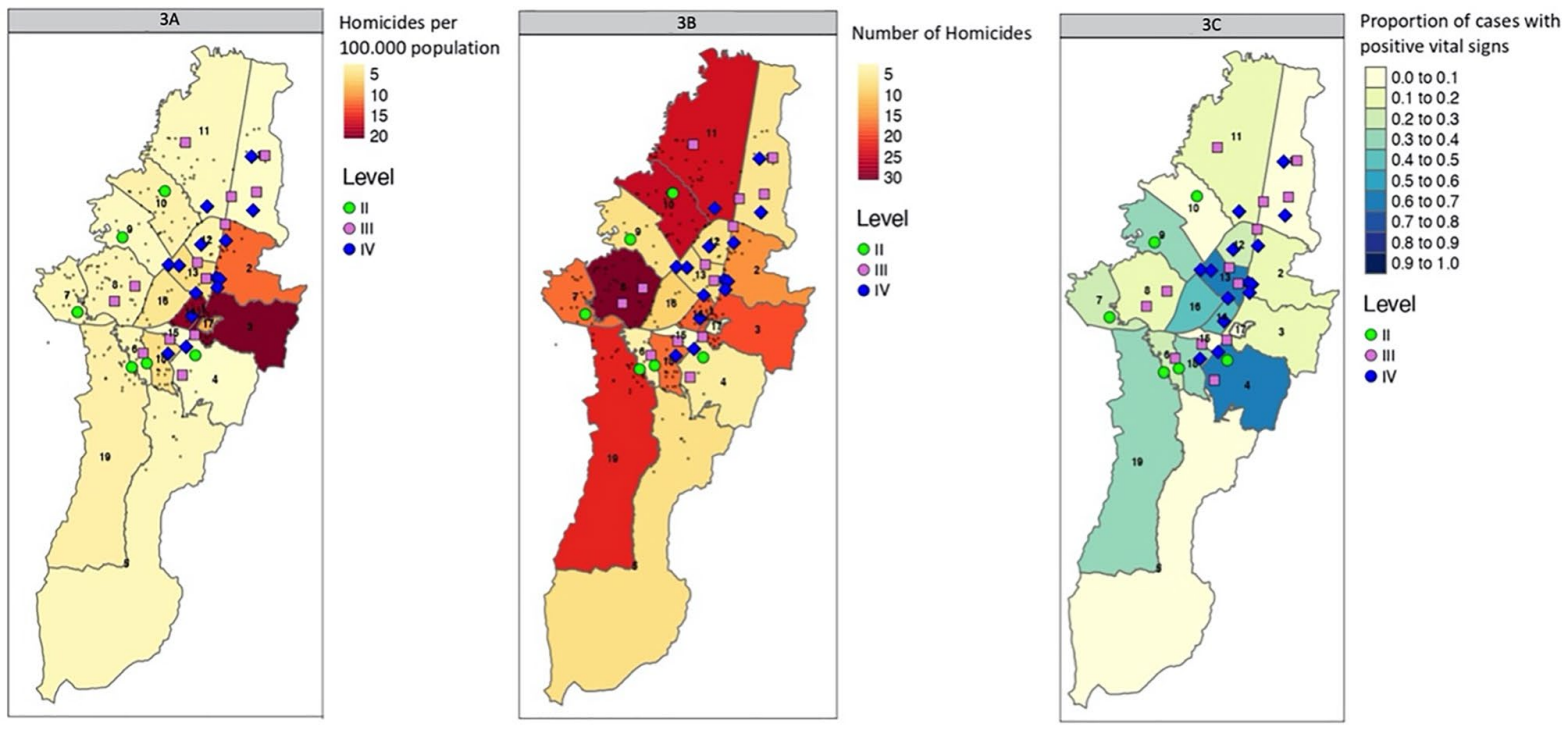
Relation between administrative localities, medical facilities, and number of homicides. Georeferencing. (**A**). Rate of homicides per 100.000 population. (**B**). Number of homicides per locality. (**C**). Proportion of PCI cases that arrived with vital signs to the emergency room per locality. Hospital facilities are represented by its different levels of care, II: Green, III: Pink, IV: Blue.

We used the 20 administrative localities of Bogotá to georeference the data. As illustrated in Fig. 3A, locality 3 (20.2 per 100,000), 14 (19.3 per 100,000), and 2 (11.8 per 100,000) have the highest PCI rates in the city. However, when considering the locality 8 had the highest amount with 30 (12.3%), followed by locality 10 with 25 (10.3%), and locality 11 with 24 (9.9%) (Fig. 3B). Figure 3C shows the number of cases in every locality that arrived with vital signs at a medical facility. The localities with the highest proportion that arrived at a medical facility were number 13 with 0.57, number 4 with 0.4, and 14 with 0.33. Georeferencing is illustrated in Fig. 3.

### Classification and regression tree

**Figure 4:**
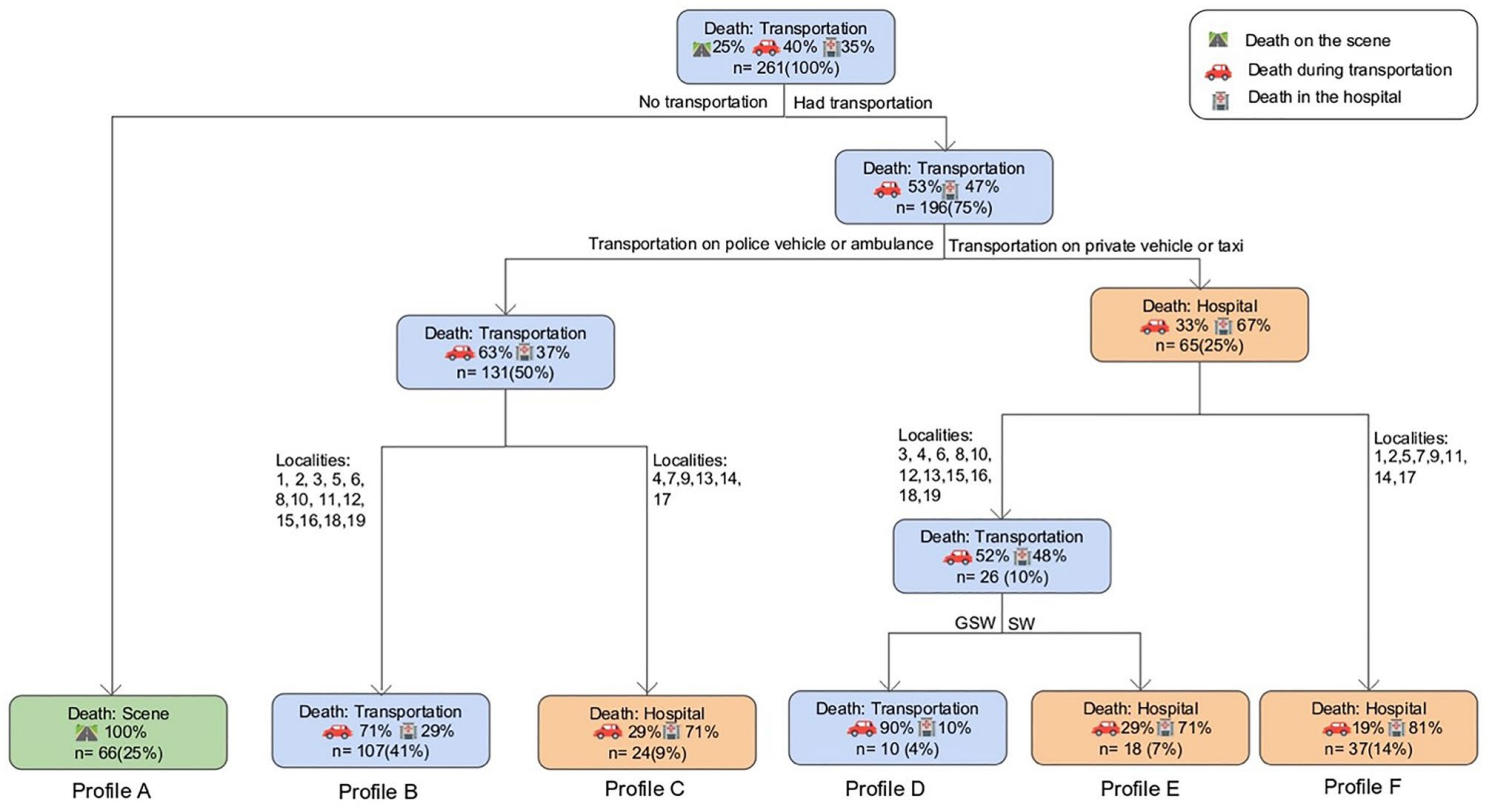
Classification and Regression Tree. GSW (Gunshot wound). SW (Stab wound).

CART analysis obtained six victim profiles that describe conditions where the events leading to the victim’s death were most likely to occur. Figure 4 illustrates the results. The variables with the highest relevance values were the average time of transportation (80.3%), localities (12.5%), injury mechanism (4.5%), and age (2%).

Profile A shows that among victims who did not have access to transportation, 66 (25%) died at the scene. Thus, the lack of means of transportation determined the place of death. The cases of profiles B and C were transported by ambulances and police vehicles, but the locality where the victims were injured determined their deaths. If the trauma occurred in one of the city’s six central localities, the victim had a higher probability of reaching a medical facility, as seen in 24 cases (9%) (Profile C); however, if the homicide occurred at any one of the peripheral localities, the patient was more likely to die during transportation, as seen in 107 cases (41%) (Profile B). The remaining profiles were transported by taxi or private vehicles. If the trauma occurred at 8 of the central localities, the patients were more likely to die at the hospital**,** as seen in 37 cases (14%) (Profile F). Patients suffering from GSWs at a peripheral locality were more likely to die during transportation, as seen in 10 cases (4%) (Profile D), while patients suffering SWs were more likely to die at the hospital, as seen in 18 cases (7%) (Profile E).

### Gun shots wounds compared to stab wounds

In cases injured by GSWs compared with SWs, the time from injury to death was shorter (30.38 SD, ±52.1 201.64 SD, and ±963.8 respectively), with a statistically significant difference (*p* = 0.002). Patients with GSWs and registrable vital signs, of which there were 13 cases (15.8%), arrived less frequently at the ER compared with SW injuries, of which there were 38 cases (33.6%) (*p* = 0.008). Associated injuries were more frequent in cases of GSW than in victims with SWs (98.2% vs 62%) (*p* < 0.001) (Table 2).

A significantly higher proportion of patients who sustained a GSW showed right atrium compromise compared to those who sustained a SW (40.5% vs 11.2%, respectively) (Table 2); additionally, injury of more than one heart chamber was more likely after a GSW compared with a SW with a statistically significant difference (*p* value < 0.001 for both comparisons) (Table 2).

Patients with more than one cardiac chamber compromised had a significantly higher likelihood of dying outside a medical facility (*p* 0.001). Additionally, patients with head or face injuries were also more likely to die outside a hospital than in a medical facility (*p* = 0.037). (Table 4).

**Table 4:** Comparison of Characteristics between 2007 and 2017.

Characteristic	2007	2017	*p *value
	n = 100 (100%)	n = 214 (100%)	
Male, sex n(%)	91 (91)	205 (96)	0.149
Age in years, mean (SD)	28.4 (10.25)	29.3 (9.73)	0.541
Aggressor, n(%)			
Authority	1 (1.3)	1 (1.3)	0.011
Criminal group	0 (0.0)	7 (9.5)	
Known	21 (27.6)	28 (38.3)	
Unknown	54 (71.0)	37 (50.6)	
Motive, n(%)			
Fight	27 (48.2)	41 (50.6)	
Robbery	18 (32.1)	19 (23.4)	
Revenge	6 (10.7)	16 (19.7)	0.101
Police operation	3 (5.3)	0 (0.0)	
Other	2 (3.5)	2 (2.4)	
Family violence	0 (0.0)	3 (3.7)	
Wound mechanism, n(%)			
GSWs	51 (51.0)	97 (45.3)	0.414
SWs	49 (49.0)	117 (54.6)	
Victims drug consumption, n(%)			
Alcohol	37 (37.0)	109 (50.9)	
Non consumption	60 (60.0)	93 (43.4)	0.021
Others	3 (3.0)	12 (5.6)	
Death place, n(%)			
Scene	42 (45.1)	54 (25.2)	
Transportation	32 (34.4)	93 (43.4)	0.002
Medical facility	19 (20.4)	67 (31.3)	
Transportation mean, n (%)			
Ambulance	3 (3.5)	2 (1.0)	0.005
Taxi	5 (5.9)	18 (9.1)	
Police vehicle	26 (30.9)	93 (47.4)	
Private vehicle	7 (8.3)	27 (13.7)	
Other	1 (1.1)	2 (1.0)	
No transportation	42 (50.0)	54 (27.5)	
Time wound-ER in minutes, mean (SD)	20.05 (13.70)	23.11 (17.32)	0.492
Vital signs at admission, n(%)			
Yes	18 (19.7)	38 (17.7)	< 0.001
No	31 (34.0)	122 (57.0)	
Death on the Scene	42 (46.1)	54 (25.2)	
Type of medical attention, n(%)			
Prehospital care	1 (1.0)	1 (0.4)	0.111
Emergency room care	4 (4.3)	27 (12.6)	
Surgical care	15 (16.1)	40(18.6)	
Death before admission	73 (78.4)	146 (68.2)	
Initial medical center level, n(%)			
1st (Lowest)	6 (6.5)	21 (9.8)	0.003
2nd	13 (14.1)	25 (11.6)	
3rd	30 (32.6)	103 (48.1)	
4th (Highest)	1 (1.0)	11 (5.1)	
Death on the scene	42 (45.6)	54 (25.2)	
Definitive medical center level, n(%)			
1st (Lowest)	3 (3.2)	14 (6.5)	0.001
2nd	13 (14.1)	23 (10.7)	
3rd	33 (35.8)	109 (50.9)	
4th (Highest)	1(1.0)	14(6.5)	
Death on the scene	42 (45.6)	54 (25.2)	

GSWs, Gunshot wounds; SWs, Stab wounds; ER, Emergency room.

### Comparison between 2007 and 2017

The 2007 and 2017 populations had similar gender distributions and average ages. The outcomes were similar in terms of homicide characteristics, motive, and injury mechanisms. However, there were some differences as the victim's prior alcohol consumption, significantly higher in 2017 (*p* < 0.05). Regarding variables related to medical attention, the data showed that in 2007, 42 (45.1%) victims died on the scene, while a lower percentage died in 2017, as this number reached 54 victims (25.2%) (*p* = 0.002). Other aspects related to medical attention, such as the vehicle, time of transportation, vital signs upon admission, type of medical care, and initial medical center level, did not have a statistically significant difference, as shown in Table 4.

## Discussion

The review of autopsy reports conducted in 2017 by the INMLCF enabled us to identify 261 cases of PCI in the city, which is a considerably high number of cases when compared with the available literature^5,22^. Traditionally, PCI characterization is performed in patients that arrive at a medical facility, leaving aside the victims who died at the scene or during transportation. However, we identified sociodemographic factors and trauma characteristics that determine if victims will eventually reach medical care and their likelihood of survival. Recognizing and analyzing these characteristics and factors is of paramount importance to public health policies and medical care.

On the other hand, when comparing our data to 2007, we identified differences regarding the victim's prior alcohol consumption, significantly higher in 2017, and that a significantly lower percentage of victims died at the scene in 2017 (25.2% vs 45.1%). These findings support the concept that the solution to the high mortality of PCI depends in large part on sociocultural changes, and of course better trauma alert and care systems.

Regarding general characterization, our sample shows a clear predominance of young male adults as the principal victims of PCIs, accounting for 94.6% of cases. These demographic features are in line with previous reports in the existing medical literature^10,11,13,16–19,23–36^. Naughton and colleagues found that 69% of PCI cases occurred during March and August, with 62% of the patients suffering the trauma on a Thursday, Friday, or Saturday. Furthermore, most homicides occurred during weekends and holidays, during brawls or assaults, and 83% of the cases occurred between 3:00 pm and 3:00 am^28^. In our sample, the months with the highest rates of cases were January, December, and April, and the days with the highest rates of cases were Saturday, Sunday and special days. Additionally, late hours (20:00–22:00) registered the most cases, and brawls were reported as the most common scenario. In this sense, our data are concordant to Naughton et. al. It is important to recognize that alcohol consumption plays a role in victimization. While it has been stated that the influence of alcohol has not been demonstrated to increase death rates, alcohol does affect mortality because a patient may be less capable of defending themselves or calling for help under the influence^18,28^. Additionally, we identified the months, days, and hours with higher PCI rates, and we believe that during these periods, implementing prevention strategies and police enforcement should be prioritized.

Considering literacy, Caycedo et al.^25^ described that their sample was mainly composed of unemployed individuals, convicts, or outcasts. Naughton et al.^28^ characterized their sample as follows: 62% were blue-collar workers, 8% were white-collar workers, and 27% were unemployed. Our research shows that victims are also young males, unemployed and have low levels of literacy, with the highest degree being a high school diploma. These data contrast the victim's profile, and concordantly matches the features previously reported in homicide-related studies, where the victims are often young men with low education and high alcohol consumption^2,8,9,37^. We encourage that education campaigns should target this population.

Another critical factor was availability of transportation. The CART model reveals that having transportation available is essential for determining the place of death and access to medical care. Unconventionally, police vehicles were the primary means of transportation, carrying almost half of the victims to a medical facility, thus establishing the police force as the primary first responders in PCI cases. Molina et al.^27^. found that, in Philadelphia, being transported by the police was a statistically significant predictor of survival; transportation by the local fire department and private vehicles was also reported. On the contrary, Naughton and colleagues state that in Alabama, victims are predominantly transported in ambulances or air lifted^28^.

The above evidence demonstrates how medical literature shows the varying types of primary first responders in PCIs and its relation to victims’ prognosis.

Time of transportation is another key factor. Even though we report that transportation took an average of 24 ± 16 min, this information only corresponds to 53.3% of all transports, as taxi drivers and private vehicles did not keep an official time record. Nevertheless, we obtained indirect data that supports that time is a crucial factor. First, the geographic analysis proved that the localities in midtown had a higher concentration of third and fourth level medical centers and consequently, patients arrived with vital signs. Second, the multivariate analysis showed that private vehicles and taxis were more likely to have transported the victim with vital signs to the emergency room. Third, the CART model evidenced that being transported by taxi or a private vehicle expands the range of localities where the patient can access medical care with vital signs, receive treatment, and eventually die. Although police vehicles and ambulances may have more skilled personnel to attend to emergencies, waiting for these vehicles can cost valuable minutes. Therefore, reducing the time-distance relationship is crucial to increasing the victim's access to surgical treatment^38,39^.

Gervin and colleagues stated that a speedy transfer to an appropriately equipped medical center, rather than stabilizing on the field, was a predictor of survival^31^. Likewise, Mina et al.^13^ evidenced a statistically significant difference in the mortality ratio when victims were transported in less than 10 min compared to those transported in more than 10 min. Other case series support the notion that the time of transport is an essential survival factor^24,28,29,31,32,39–41^.

Contrasting transportation availability in low-middle income countries, an exploratory study in patients with traumatic brain injury (TBI) located in Salvador de Bahía (Brazil) revealed that territory clusters where health services, goods and higher education are concentrated often defer from clusters were TBI occurs^42^. It was much worse for indigenous people in Rabinal, Guatemala, where it was reported that availability for transportation, among other economic and cultural barriers, constituted the hardest difficulties to reach a health care facility^43^. Data reveals that there are deep structural inequities throughout the territory which can be summarized as inefficient transportation and poor distribution of high-level care facilities.

Regarding trauma characteristics, we identified that 23% of cases were injured outside the limits of the cardiac box. Almost half of the fatal victims sustained SWs and half sustained GSWs. The current analysis and the previous literature indicates that GSWs are more lethal than SWs^10,13,16–18,23,24,24–30,40,41,44^. Our data shows that GWs are associated to 2 or more compromised cardiac cavities (52% of the cases with a statistically significant value), with more associated lesions and less time available to reach a medical facility. These findings support the evidence that GWs are more lethal than SWs, and they are consistent with the local hospital succession of operated and recovered patients, most of which suffered SWs^16,23^.

According to Asencio et al., the ventricular chambers are the most frequently injured, and the right ventricular chamber is the most frequently compromised, as observed in 52% of the cases^12,44,45^. However, there is a discrepancy between the in-hospital studies and autopsy studies. Autopsy studies report that the most frequently injured chamber is the left ventricle chamber with an incidence of 31–42%^18,22^. Our study shows that the right ventricular chamber was the most injured in 48.6% of the cases, followed by the left ventricular chamber with 47.49%. Furthermore, SW injuries were associated with only one compromised chamber (*p* < 0.001) while GSWs were associated with two or more compromised chambers (*p* < 0.001) Grades IV and V of severity were strong predictors of mortality.

Regarding the comparison of data sets between 2007 and 2017, the socio-economic context in Bogotá improved between those 2 years. The poverty index dropped from 19.7 to 12.4%. The GINI index went from 0.533 to 0.498 points, and the gross domestic product per capita increased from $16,027,709 (7720 USD) to $30,307,765 (10,270 USD)^46^. Nevertheless, the factors contributing to the materialization of homicides have not improved in 10 years. In 2017, victim alcohol consumption increased alarmingly. On the bright side, medical care following the trauma has improved. While 42 (50%) cases could not access any type of transportation in 2007, this was only true for 54 (27.5%) of the patients suffering from a PCI in 2017. Police vehicles went from transporting 26 (30.9%) victims in 2007 to 93 (47.4%) in 2017. Unfortunately, the time of transportation was longer in 2017 without a statistically meaningful difference (*p* = 0.492); however, said difference could have impacted arrival to the ER with vital signs on admission, which was higher in 2007 with 18 (19.7%) cases than in 2017 with 38 (17.7%) cases (*p* < 0.001). We consider that this improvement might be due to better warning systems and faster responses. Even though the city's statistics regarding the poverty index and response time have improved, the factors surrounding the traumatic event have slightly changed but have not necessarily improved.

The complexity of the factors involved in the prognosis of these cases and our research results invite us to develop surveillance, communication, and computer systems by area, integrated with individual sensors of health conditions, and maintain automated and cooperative monitoring systems. It would be ideal to detect emergency situations at the immediate moment of their occurrence, and even activate unmanned transport systems to improve accessibility to the required care, as suggested by Chandramohan et al.^47^.

Regrettably, the teams collecting information in 2007 and 2017 were different; there is no record of information collected for November, December, and January 2007, thus limiting any further statistical analysis. This study is also limited by its retrospective nature, and the fact that the results obtained only apply to Bogotá. Additionally, there are still gaps to fill, such as determining the burden of PCIs along with the impact on the working force lost for the young male patients, among other variables. Nevertheless, our study is the first to integrate the anatomical and demographic factors of PCI victims and provide evidence of how homicides may vary depending on socio-urban factors; this is a critical tool when identifying problems and finding solutions. We leave behind a more rigorous database for future investigations because we believe that the injuries intended to cause an individual's death, like PCI, which provides a guideline to analyze interpersonal injuries in our society.

## Conclusions

PCIs are the most lethal injuries globally and its mortality relies on multiple factors. Young, low-scholar males are by far the most affected population. Holidays and scenarios where alcohol consumption is normalized are tightly related to this type of injury. Regarding sociodemographic factors, being injured far from high-level institutions and delayed transportation time are factors related to high mortality. Two or more compromised cavities and severe trauma are highly lethal as well. PCI injuries can occur even when they are outside the cardiac box limits. We believe that to mitigate the high impact of PCIs, precautions must be taken by first responders, either through establishing faster response times or by providing a straight referral to an institution with proper surgical resources to treat the patient. We also believe that the city's public health authorities must consider building specialized medical centers on the city's outskirts and distributing new facilities more homogenously throughout the territory to improve the chances of survival of these patients.

## Methods

A panel-type cross-sectional study was designed using the HIPPA guidelines, the Declaration of Helsinki’s ethical principles, the current legislation on research (Res. 008430-1993 and Res. 2378-2008), the International Committee Rules for Medical Journal Editors (ICMJE) and the authorization from INMLCF (0329-EML-SIC-2018). The institutional review board from Universidad del Rosario approved the protocol in 2018 (DVO005-1-349-CEI925).

In Colombia, the only institution authorized to perform autopsies in trauma cases is the INMLCF; therefore, INMLCF and Universidad del Rosario established an agreement to analyze data in 2018. Additionally, to compare the homicide characteristics of penetrating cardiac injuries between 2007 and 2017, Pedraza et al. authorized the authors to access and analyze their database, but it had limitations. Their database lacked details of the victim's occupation, education, residence zone, and if the trauma occurred during holidays. Additionally, they could not gather autopsies made during November, December, and January.

### Data collection

We accessed the physical case files of all the violent deaths occurring in Bogotá throughout 2017. Each case file contained three reports: the police report, the medical history, and the autopsy drafted by the INMLCF. The police report provided information on the victims' demographic background, the details surrounding the homicide, and the actions taken by the police. But, details were incomplete when they found the victim alone. The medical history described the characteristics of the trauma and the medical care provided if the victim arrived at a hospital facility. Finally, the autopsy report contained an anatomic description of the trauma and a toxicology blood analysis. We collected variables in each of these reports.

In 2017, the INMLCF wrote 4110 autopsy reports. INMLCF provided a list of 1505 cases labeled with a different diagnosis as the cause of death: "hypovolemic shock," "acute anemia," "cardiogenic shock," "hemorrhagic shock," or "polytrauma". We identified 273 cases of PCIs, and excluded one suicide case (n = 1) and eleven (n = 11) were unavailable since they were being processed by the court, leaving a total of 261 cases. Variables were collected manually using Microsoft Excel. Other authors reviewed twenty-five percent of the data to identify collection bias.

### Statistical analysis

The data were analyzed using R Software 3.6.3. Quantitative variables are presented as means and standard deviations, and qualitative variables as frequencies and percentages. We also performed a bivariate analysis to identify possible factors associated with Injury mechanism and status and admission as well as to assess differences between data from years 2007 and 2017. For this purpose the Kruskal–Wallis test, Chi-square test, and Spearman correlation coefficient were calculated accordingly.

In order to explore more deeply the factors associated with place at death, we use Classification and Regression Trees^48^ as implemented in Rpart R package^49^ as an exploratory tool to uncover complex interactions between selected covariates and the place at death. This method has been used before successfully by one of the authors to explore associations on outcomes of interest in the context of observational studies (see for example^50–52^).

Finally, georeferencing maps were done using R Software 3.6.3 and Google Maps. Coordinates of homicide and trauma hospitals were obtained by Google Maps and introduced in R Software 3.6.3. With the data, a heat map on homicide cases, rates per locality, and the rate of arrival to the emergency room with vital signs per locality were graphed.

### Anatomical definition of the cardiac box and graphic analysis

For this research, we defined the limits of the cardiac box as follows: the superior border, the line across the clavicles and the sternal notch; the inferior border as the line across the lower costal margin; the left lateral margin as the left anterior axillary line; the right lateral border as the right parasternal line.

We constructed a graphical analysis of the cardiac box based on Netter's^53^ illustration of the thorax and placed a grid over the margins of the cardiac box. We identified the injuries' location by reading the autopsy reports and marking the entrance hole.

We further tailored a classification and regression tree (CART) to assess the predictive power of relevant socio-demographics, homicide characteristics, and primary attention variables to forecast the place of death. Those variables included age, sex, death mechanism, means of transportation, prior drug use, and the localities where the homicide occurred. The algorithm quantified each variable's prediction power and assigned the relevant value. Then, it created a tree figure to build different profiles that predicted the victim's place of death.

Finally, we drafted the manuscript following the Strengthening the Reporting of Observational Studies in Epidemiology (STROBE) statement^54^.

### Ethics approval and consent to participate

The institutional review board from Universidad del Rosario approved the research protocol in 2018 (DVO005-1-349-CEI925). The need/requirement for informed consent was waived by the Research Ethics Committee of the Universidad del Rosario—life science division, authorized by the National Institute of Legal Medicine and Forensic Sciences owner of the files of the analyzed cases.

## Data Availability

The datasets generated and/or analyzed during the current study are not publicly available due to autopsies privacy restrictions but are available from the corresponding author on reasonable request.
